# Ventromedial hypothalamus relays chronic stress inputs and exerts bidirectional regulation on anxiety state and related sympathetic activity

**DOI:** 10.3389/fncel.2023.1281919

**Published:** 2023-12-14

**Authors:** Jie Shao, Yan Chen, Dashuang Gao, Yunhui Liu, Nan Hu, Lianghong Yin, Xinzhou Zhang, Fan Yang

**Affiliations:** ^1^Department of Nephrology, The Second Clinical Medical College, Jinan University (Shenzhen People’s Hospital), Shenzhen, China; ^2^The First Affiliated Hospital, Jinan University, Guangzhou, China; ^3^The Brain Cognition and Brain Disease Institute, Shenzhen Institute of Advanced Technology, Chinese Academy of Sciences, Guangdong Provincial Key Laboratory of Brain Connectome and Behavior, CAS Key Laboratory of Brain Connectome and Manipulation, Shenzhen-Hong Kong Institute of Brain Science-Shenzhen Fundamental Research Institutions, Shenzhen, China; ^4^School of Sport Medicine and Rehabilitation, Beijing Sport University, Beijing, China; ^5^Department of Nephrology, Shenzhen People’s Hospital (The Second Clinical Medical College, Jinan University, The First Affiliated Hospital, Southern University of Science and Technology), Shenzhen, China

**Keywords:** chronic stress, ventromedial hypothalamus, anxiety, sympathetic nervous system, EPSC (excitatory postsynaptic current)

## Abstract

Chronic stress can induce negative emotion states, including anxiety and depression, leading to sympathetic overactivation and disturbed physiological homeostasis in peripheral tissues. While anxiety-related neural circuitry integrates chronic stress information and modulates sympathetic nervous system (SNS) activity, the critical nodes linking anxiety and sympathetic activity still need to be clarified. In our previous study, we demonstrated that the ventromedial hypothalamus (VMH) is involved in integrating chronic stress inputs and exerting influence on sympathetic activity. However, the underlying synaptic and electrophysiological mechanisms remain elusive. In this study, we combined *in vitro* electrophysiological recordings, behavioral tests, optogenetic manipulations, and SNS activity analyses to explore the role of VMH in linking anxiety emotion and peripheral SNS activity. Results showed that the VMH played an important role in bidirectionally regulating anxiety-like behavior and peripheral sympathetic excitation. Chronic stress enhanced excitatory inputs into VMH neurons by strengthening the connection with the paraventricular hypothalamus (PVN), hence promoting anxiety and sympathetic tone outflow, an important factor contributing to the development of metabolic imbalance in peripheral tissues and cardiovascular diseases.

## Introduction

The central nervous system (CNS) plays a crucial role in regulating peripheral physiological processes in response to various emotional states (Critchley and Harrison, 2013). For instance, anxiety can lead to accelerated heart rate and elevated blood pressure through the activation of the sympathetic nervous system (SNS) (Hu et al., 2019; Bigalke et al., 2023; Hsueh et al., 2023). The adrenal glands, which are innervated by the SNS, are responsible for releasing stress-related hormones such as norepinephrine (NE) and cortisol (Schibler and Brown, 2005; Waxenbaum et al., 2023). Together with direct innervation of autonomic nerves, these hormones elicit various stress responses, such as heightened heart rate, increased respiration rate, elevated blood pressure, and increased glucose levels, enabling the body to cope with imminent threats (Dos Reis et al., 2014; Yaribeygi et al., 2017). Typically, the SNS response is triggered to prepare the body for potentially dangerous situations or acute stress. Once the stressors subside, the SNS generally returns to the relax state (Payne et al., 2015). However, prolonged and repeated exposure to stress can disrupt the recovery processes and induce an overactivated state (Dallman, 1993; Holwerda et al., 2018). Overactivation of the SNS is implicated in the development of several metabolic and cardiovascular diseases, including obesity, diabetes, and hypertension (Converse et al., 1992; Canale et al., 2013; Nguyen et al., 2014). Chronic stressors are critical contributors to the excessive excitation of the SNS through stress-coping circuits in the CNS (Asakura et al., 2000; Anthony et al., 2014; Elsaafien et al., 2021).

The CNS stress-response circuitry, comprising integral components such as the ventromedial hypothalamus (VMH) and paraventricular hypothalamus (PVN) (Anthony et al., 2014; Henckens et al., 2016; Yuan et al., 2019; Shao et al., 2022) perform an essential role in transmitting and processing stress-related information, as well as modulating peripheral SNS activity and regulating hormonal secretion via the hypothalamic-pituitary-adrenal (HPA) axis (Shenton and Pyner, 2016; Iigaya et al., 2017; Yang et al., 2020; Liu et al., 2023). Despite these critical functions, the definitive neuroanatomical locus mediating chronic stress-induced SNS hyperactivity, along with the corresponding neurobiological mechanisms, remains poorly understood. The VMH, especially its steroidogenic factor-1 (SF-1)-expressing neuronal subpopulation, is strongly associated with affective disorders such as anxiety and fear (Silva et al., 2013; Kunwar et al., 2015; Anderson, 2016; Viskaitis et al., 2017). Studies have shown that VMH SF-1 neuronal activity is markedly enhanced in response to anxiety-like behavior following predator stress exposure (Kennedy et al., 2020), while blockade of VMH glutamate signaling can effectively ameliorate anxiety (Cheung et al., 2015; Li et al., 2023), suggesting that the VMH is involved in the regulation of stress-coping behavior. The VMH also serves as a vital hub linking emotional states and peripheral physiological homeostasis (Fioramonti et al., 2009; Kuperman et al., 2010; Anderson, 2016; Viskaitis et al., 2017). Recent studies from our research group revealed profound electrophysiological and molecular changes within the VMH following chronic stress, precipitating anxiety-like emotional states and disruptions in peripheral metabolic equilibrium (Yang et al., 2020; Shao et al., 2022). For example, chronic stress over a 4-week period has been shown to enhance projections from the bed nucleus of the stria terminalis (BNST) to the VMH, thereby provoking anxiety-like behavior and specifically affecting bone loss (Yang et al., 2020). The SNS has also been implicated to transmitting activity changes in the VMH during chronic stress exposure and subsequently disrupting metabolic homeostasis in peripheral tissues (Yang et al., 2020; Liu et al., 2023). Based on these studies, we hypothesized that there is a tight correlation between chronic stress-induced anxiety and peripheral SNS activity through neuronal activity of VMH neurons.

To test this hypothesis, we combined electrophysiological recording, behavioral test, optogenetics manipulation and SNS activity analyses, and found there is a negative correlation between anxiety-like behavior and stress-hormone in peripheral serum, including NE, cortisol and renin, which represented activity of SNS (Hu et al., 2020), HPA (Young et al., 2021) and RAAS (Renin-angiotensin-aldosterone system) (Ames et al., 2019), respectively. Bidirectional manipulation of VMH neuronal activity induced opposite changes of SNS activity and also anxiety-like behavior. Chronic stress could increase the excitatory input of VMH neuron and increase excitability via glutamate receptors to exerts influence on SNS, and excitatory input from PVN (origin of HPA axis) might be main source of stress-related information into VMH during chronic stress. These results indicated that the VMH is a critical hub that maintain anxiety state and affect the SNS excitability after chronic stress.

## Materials and methods

### Animal and chronic stressor treatments

Male C57BL/6 wild-type mice were purchased from the Guangdong Medical Laboratory Animal Center (Guangzhou, China) and male SF1-Cre mice (Jackson stock no: 012462) were obtained from the Jackson Laboratory. All mice were randomly assigned to either control or stress groups (7 mice each group). Those in the stress group (aged 4 weeks) underwent daily exposure to a random stressor – either wet bedding, tube restraint, or random squeezing – for a continuous period of 28 days. All stressed mice were subjected to stressors with same protocol. The control group mice were not subjected to any stressor. Mice were housed at a temperature of 22–25°C under a 12-h light-dark circadian cycle (lights on at 7:00 a.m. and off at 7:00 p.m.) with *ad libitum* access to food and water. All procedures were carried out in accordance with the protocols approved by the Ethics Committee of the Animal Care and Use Committee of Shenzhen People’s Hospital (AUP-220920-SJ-0561-01) and Shenzhen Institutes of Advanced Technology, Chinese Academy of Sciences (SIAT-IACUC-190219-NS-YF-A0582).

### Behavioral tests

#### EPM

To assess anxiety-related behaviors after chronic stress, mice in both the stressed and wild-type groups were placed in the center of a maze, with behavior then recorded for 5 min using an overhead-mounted camera. Video recordings from the EPM test were analyzed using AnyMAZE software (Stoelting Co., Wood Dale, USA) to acquire data such as time spent in the open and closed arms and number of entries into the open arm. For light stimulation, blue (20 Hz, 10 ms) or yellow (constant) light was applied through silica optical fiber during the whole test (5 min).

#### OFT

For this test, mice were placed in the center of an open field, with behavior then recorded for 10 min. As above, videos were analyzed to extract data related to resident time and entries into the center area. For optogenetic manipulation, blue (20 Hz, 10 ms) or yellow (constant) light was applied during the whole test (10 min).

### Slice preparation

Mice were deeply anesthetized with isoflurane and rapidly decapitated. Brain tissues were removed and transferred into ice-chilled cutting solution containing (in mM): choline chloride 110; KCl 2.5; Na-ascorbate 1.3; Na-pyruvate 0.6; MgCl2 7.0; CaCl2 0.5; NaH2PO4 1.3; NaHCO3 25; and glucose 20 (pH 7.4), bubbled with 95% O2 and 5% CO2 for at least 30 min before use. Coronal slices (250–300-mm thick) were prepared on a Vibratome VT1200 (Leica, Wetzlar, Germany), and incubated in artificial cerebrospinal fluid (ACSF) containing (in mM): NaCl 125; KCl 2.5; Na-ascorbate 1.3; Na-pyruvate 0.6; MgCl2 1.3; CaCl2 2.0; NaH2PO4 1.3; NaHCO3 25; and glucose 10 (pH 7.4), bubbled with 95% O2 and 5% CO2 at 37°C for 30 min before use. The slices were equilibrated in ACSF at room temperature (26°C) for at least 40 min before being transferred to the recording chamber. During the entire recording procedure, the recording chamber was perfused with ACSF bubbled with 95% O2 and 5% CO2 at room temperature. Any administered drugs were also applied using perfusing ACSF.

### Electrophysiology

The VMH was identified based on landmarks (3rd ventricle) or mCherry protein fluorescence. Sealing and patching were performed with multi-clamp 700B amplifiers (Molecular Devices, San Jose, USA) under visual guidance with a Nikon FN1 microscope (Tokyo, Japan). Electrophysiological signals were digitized at 10 kHz using Digidata 1440A (Molecular Devices, San Jose, USA) and analyzed using Clampfit v11.2 software. Whole-cell recordings were performed with borosilicate glass electrodes (0.69 mm OD, 5–7 MΩ), with the internal solution containing (in mM): K-gluconate 135.0; KCl 4.0; NaCl 2.0; HEPES 10; EGTA 4.0; Mg-ATP 4.0; and Na-GTP 5.0. Osmolality was adjusted to 300 (± 10) mOsm kg-1 with sucrose and pH was adjusted to 7.4 with KOH.

After forming a high-resistance seal (> 1 GΩ) and breaking the membrane, the cell was held in voltage-clamp mode for 5 min until access resistance stabilized. The cells were held at −70 mV to record spontaneous EPSCs and mEPSCs, tetrodotoxin (TTX, 1 μM) was applied by ACSF perfusion during the mEPSCs recording. To verify excitatory projections from the PVN to VMH, we applied blue light illumination during the evoked EPSC recordings of VMH neurons. Antagonists of glutamate receptors (d-APV and NBQX) were perfused with ACSF to confirm excitatory connections.

### Stereotaxic surgery and viral injection

For all stereotaxic surgeries, 12–16-week-old mice were anesthetized by an intraperitoneal injection of pentobarbital sodium (0.2% in saline, 1 mL/100 g). The stereotaxic surgical procedures were performed based on previous research (Liu et al., 2021). To target the VMH, injection coordinates relative to Bregma: antero-posterior −1.58 mm, mediolateral ± 0.3 mm, and dorsoventral −5.5 mm. For the PVN, the injection coordinates relative to Bregma were antero-posterior −0.80 mm, mediolateral ± 0.37 mm, and dorsoventral −5.0 mm. Unless stated otherwise, 0.25 μL of viral vector was used to inject into the VMH or PVN at a rate of 0.1 μL/min using a 10-μL Hamilton syringe and a syringe infusion pump (World Precision Instruments, USA).

### Microdialysis

After anesthetization via intraperitoneal injection of pentobarbital sodium (0.2% in saline, 1 mL/100 g), mice were placed and fixed in a stereotaxic frame. A microdialysis probe (Bioanalytical System Inc., Indiana, USA) was implanted into the VMH of each mouse according to the above described coordinates. The probe was perfused with ACSF for 90 min, followed by sample collection for 30 min. Glutamate concentrations in the dialysate were determined using an enzyme-linked immunosorbent assay (ELISA) kit according to the manufacturer’s instructions.

### ELISA

The concentrations of transmitters and hormones in the collected samples of serum or cerebrospinal fluid were quantified using ELISA kits, specifically for cortisol (CUSABIO, E05113m, Wuhan, China, CSB-E05113m), NE (CUSABIO, CSB-E07870m), renin (CUSABIO, CSB-E08703m), and glutamate (Abnova, KA1670, Taiwan, China, KA1670). All experiments are performed according to the manufacturer’s instructions.

### Optogenetic manipulation

For optogenetic manipulation of SF-1 neurons in the VMH, AAV9-DIO-ChR2-mCherry, AAV9-DIO-NpHR-mCherry, or AAV-DIO-mCherry (1.0E + 12 vg/mL, Braincase, Shenzhen, China) was injected into the VMH according to the above-described coordinates. The mice were then housed for 4 weeks to allow for viral expression before conducting further experiments and data collection. A silica optical fiber (200-μm diameter, 0.37 NA. fiber with 1.25-mm ceramic ferrule; NEWDOON, Hangzhou, China) was implanted 0.2 mm higher than the viral injection site. A protective head cap was created using dental cement. The mice were allowed to fully recover before data collection. To analyze sympathetic activity, 470-nm blue light stimulation was performed at 20 Hz for 3 min in mice expressing mCherry and ChR2-mCherry, while consecutive 580-nm yellow light stimulation was performed for 3 min. Anxiety-like behavior was assessed using 20-Hz blue light or consecutive yellow light for 5 min during the EPM test or 10 min during the OFT. For functional confirmation of PVN-VMH projections, AAV-CRH-ChR2-mCherry or AAV-CRH-mCherry was injected into the PVN according to the coordinates mentioned above. After 4 weeks of expression, the mice were used for patch-clamp recordings.

### Heart rate and respiratory rate recordings

After anesthesia with isoflurane, the mice were laid on their backs with the right thigh shaved. A heating pad was used to maintain body temperature. Heart rate was recorded using a Small Animal Physiological Monitoring System (Harvard Apparatus, Holliston, USA), while ECG and respiratory rate were acquired at 1 kHz and 250 Hz, respectively. To manipulate VMH neurons, the mice were injected with the virus in advance, with 5 min of blue light (20 Hz) or yellow light (consecutive) applied during ECG recordings. Mice were anesthetized with isoflurane during the whole recording process.

### Immunohistochemistry and viral tracing

Mice were anesthetized via an intraperitoneal injection of chloral hydrate (10% in saline, 1 mL/100 g), then perfused transcardially with 0.01 M phosphate-buffered saline (PBS) and 50 mL of 4% paraformaldehyde (PFA). Brains were dissected and post-fixed in 4% PFA overnight, then transferred to 30% w/v sucrose solution to dehydrate for 48 h. Sections from the entire anterior-posterior range of the VMH were stained using a *c-fos*-specific antibody (1: 200, Cell Signaling, Danvers, USA). Briefly, sections were washed, permeabilized in 0.1% Triton X-100/PBS for 15 min/three times, washed again, and blocked in 10% normal goat serum (NGS) (w/v)/0.1% Triton X-100/PBS for 1 h. The primary antibody was added, with the sections then incubated overnight at 4°C. The following day, the sections were washed with 0.01 mM PBS, incubated with secondary antibodies (Alexa Fluor 488 goat anti-rabbit, Jackson ImmunoResearch, Pennsylvania, USA, 1:300) in 0.1% Triton X-100/PBS for 1 h at room temperature, then washed with PBS three times (5 min each). Nuclear 4′,6-diamidino-2-phenylindole (DAPI, Vector labs, Burlingame, USA, 1:5 000) staining solution was added. After staining, the slices were mounted, cover slipped with mounting medium, and observed using an Apotome (Zeiss, Oberkochen, Germany). For viral tracing, the mice were injected with AAV-CRH-mCherry into the PVN. After 4 weeks of expression, the mice were perfused, and brain tissues were collected.

### Statistical analysis

Slice electrophysiological data were analyzed using Clampfit v11.2 (Molecular Devices, San Jose, USA). The EPSC data were filtered at 2 kHz for high-frequency noise removal. All data were imported into Prism v7 (GraphPad, Boston, USA) and normality was assessed using the D’Agostino-Pearson and Kolmogorov-Smirnov tests to verify the appropriateness of the following statistical analyses. Unless stated otherwise, the data are presented as means ± standard error of the mean (SEM). Statistical significance was determined using either the two-tailed unpaired *t*-test or Whitey-Mann U test when comparing two groups, with paired *t*-tests used for the same mice before and after treatment. Multiple *t*-tests were corrected using the Sidak-Bonferroni method. A difference was considered significant at *p* < 0.05 (*p < 0.05, **p < 0.01, ***p < 0.001).

## Results

### Correlation between chronic stress-induced anxiety and increased sympathetic tone

**FIGURE 1:**
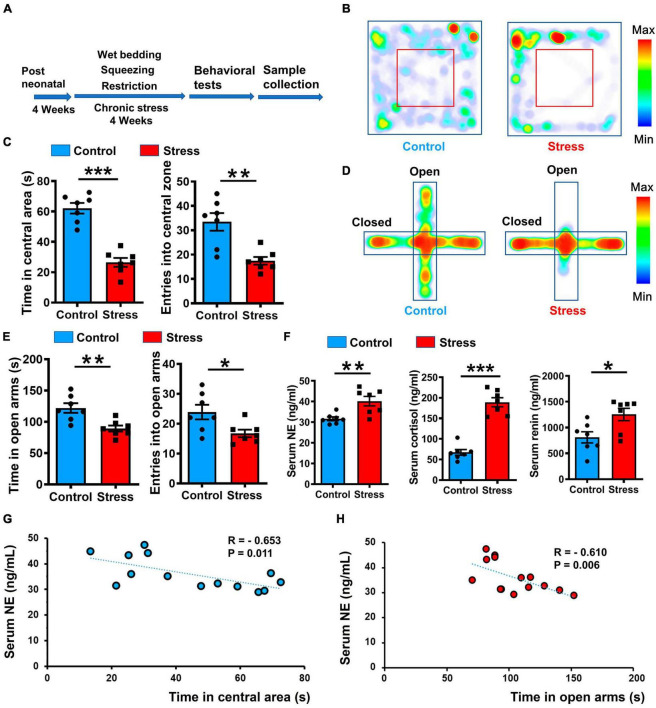
Chronic stress induced anxiety-like behavior and elevated SNS tone in mice. **(A)** Illustration of 4-week chronic stress protocol and phenotype identification. **(B)** Representative heatmaps of control (left) and stressed (right) groups in different positions in OFT. **(C)** Statistical analysis of control (blue) and stressed (red) groups in OFT, including entries into and time spent in central areas. Values represent mean ± SEM unless stated otherwise (*n* = 7 for control and stressed groups, respectively. Resident time: *p* < 0.001; Entries: *p* = 0.0016; unpaired *t*-test). **(D)** Representative heatmaps of resident time in EPM in control and stressed groups. **(E)** Statistical analysis of control and stressed groups in EPM, including entries into and time spent in open arms (*n* = 7 for control and stressed groups, respectively. Resident time: *p* = 0.0035; Entries: *p* = 0.0235; unpaired *t*-test). **(F)** Quantification of stress-related hormones in control and stressed groups based on ELISA (*n* = 7 for control and stressed groups, respectively. NE: *p* = 0.0045; Cortisol: *p* < 0.001; Renin: *p* = 0.0180; unpaired *t*-test). **(G,H)** Pearson correlation analysis between anxiety-like behavior and NE concentration, each dot represents a mouse: Left, correlation between resident time in central area of open field and NE concentration, *R* = –0.653, *p* = 0.011; Right, correlation between resident time in open arms of EPM and NE concentration, *R* = –0.610, *p* = 0.006. Data are means ± SEM, *P < 0.05, **P < 0.01, and ***P < 0.001.

Chronic stress plays a pivotal role in the development of anxiety and sympathetic overexcitation, with emerging evidence also suggesting a link between anxiety states and heightened sympathetic tone (Asakura et al., 2000; McKim et al., 2016; Yaribeygi et al., 2017; Bangsumruaj et al., 2022). Thus, we speculated that anxiety induced by chronic stress influences the potentiation of excitatory outflow. To investigate this hypothesis, we conducted a 28-day chronic variable stress procedure to induce an anxiety-like state in mice (Figure 1A). Subsequent evaluation using an open field test (OFT) revealed a marked reduction in both resident time and entries into the central zone for the stressed group compared to the control group (Figures 1B, C). In addition, the stressed mice spent less time in and made fewer entries into the open arms of the elevated plus maze (EPM) (Figures 1D, E). These findings suggest that the stressed group exhibited heightened anxiety compared to the control group. To further investigate the physiological changes associated with chronic stress-induced anxiety, we analyzed stress-related hormone levels in the serum. Notably, the stressed mice displayed elevated concentrations of NE, cortisol, and renin, suggesting an increase in sympathetic outflow (Figure 1F). Furthermore, a significant correlation was observed between the levels of NE, renin, cortisol, and the manifestation of anxiety-like behavior (Figures 1G, H; Supplementary Figure 1). These findings provide evidence supporting the close connection between the anxiety-like state induced by chronic stress and heightened sympathetic excitation.

### Activation of VMH induces anxiety-behavior and sympathetic excitation

**FIGURE 2:**
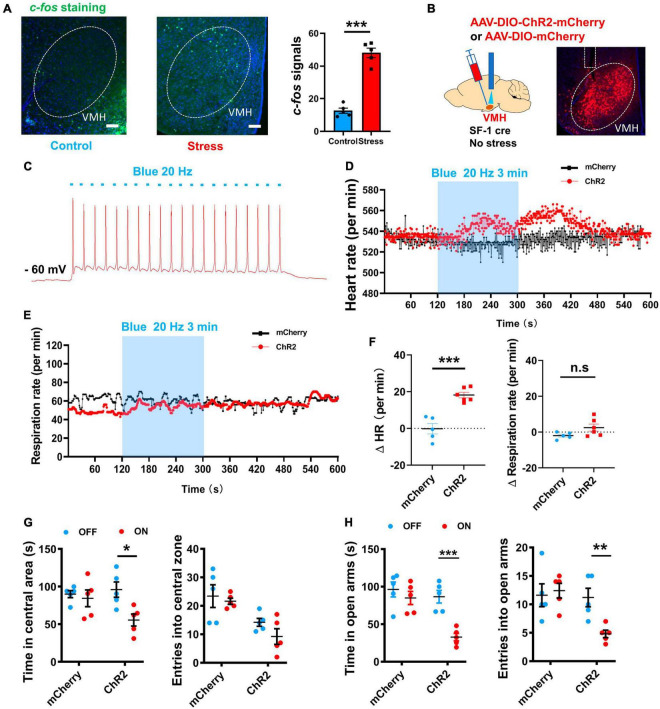
Optogenetic manipulation of VMH SF-1 neurons induced anxiety-like behavior and SNS activation. **(A)** *c-fos* staining of VMH in control and stressed groups (scale bar, 100 μm) and quantification of average amount of *c-fos*-positive cells per slice (*n* = 5 mice per group; *p* < 0.001; unpaired *t*-test). **(B)** Schematic of VMH injection of ChR2-expressing AAV viral vector to activate SF-1 neurons in SF-1 cre mice (without chronic stress treatment) *in vivo*. **(C)** Whole-cell recordings of ChR2-expressing neurons and evoked action potential firing (Blue light, 20 Hz, 20 ms). **(D)** Representative traces of heart rate change per min evoked by blue light illumination of VMH SF-1 neurons. **(E)** Representative traces of respiratory rate evoked by blue light illumination of VMH SF-1 neurons. **(F)** Statistical analysis of changes in heart and respiratory rates (per min) induced by VMH SF-1 neuronal activation (*n* = 5 mice for mCherry group and 6 mice for ChR2 group. Heart rate: *p* < 0.001; respiratory rate: *p* = 0.0825, unpaired *t*-test). **(G)** Statistical analysis of changes in anxiety-like behavior in OFT induced by 10-min blue light illumination of VMH SF-1 neurons (*n* = 5 mice for ChR2 and mCherry groups, respectively. Resident time: mCherry, *p* = 0.6544, ChR2, *p* = 0.0143; Entries: mCherry, *p* = 0.6750, ChR2, *p* = 0.1430; multiple paired *t*-test). **(H)** Statistical analysis of changes in anxiety-like behavior in EPM induced by activation of VMH SF-1 neurons (Resident time: mCherry, *p* = 0.4169, ChR2, *p* < 0.001; Entries: mCherry, *p* = 0.7466, ChR2, *p* = 0.0065; multiple paired *t*-test). Data are means ± SEM, *P < 0.05, **P < 0.01, and ***P < 0.001.

Previous studies have indicated the VMH SF-1 neurons, which serve as specific molecular markers for the dorsomedial part of the VMH (Kunwar et al., 2015), can affect sympathetic tone (Liu et al., 2023) and regulate behavioral responses during the emergence of stressors (Silva et al., 2016; Zhou et al., 2021). Our *c-fos* staining results demonstrated the activation of VMH neurons after chronic stress (Figure 2A). Based on these findings, we hypothesized that VMH activation plays a critical role in connecting anxiety and peripheral sympathetic excitation. To test this speculation, we optogenetically manipulated VMH SF-1 neurons in non-stress treated mice while monitoring heart rate data. By injecting a double-floxed inverted orientation (DIO) -based ChR2 vector into the VMH of SF-1 Cre mice implanted with a silica optical fiber, we successfully achieved *in vivo* activation of VMH SF-1 neurons to mimic activation caused by chronic stress, while maintaining real-time heart rate recordings (Figures 2B, C). Our results indicated that 3 min of activation of SF-1 neurons triggered a significant increase in heart rate, while exerting no obvious influence on respiratory rate (Figures 2D–F), suggesting a functional connection between the VMH and a specific component of the SNS. We further evaluated the effect of optogenetically activating VMH SF-1 neurons on anxiety-like behavior. The open field and EPM test results indicated an anxiety state in ChR2-expressing mice induced by blue light illumination, but not in mice injected with AAV-DIO-mCherry (Figures 2G, H). Thus, activation of VMH SF-1 neurons elicits anxiety and sympathetic activation, similar to anxiety and sympathetic overactivation after chronic stress.

### Inhibition of VMH alleviates anxiety state and decreases heart rate

**FIGURE 3:**
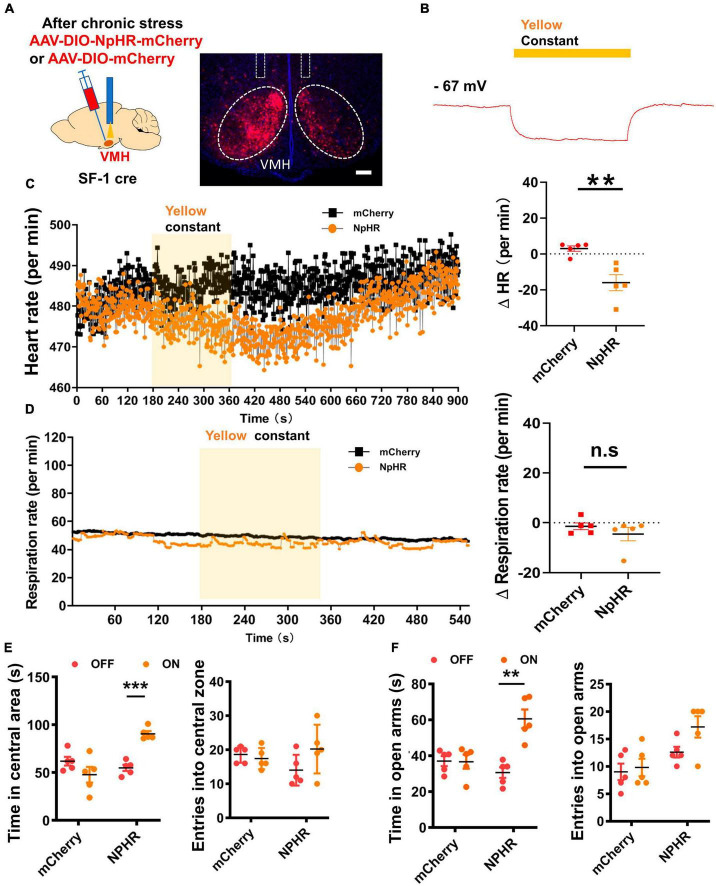
Optogenetic silencing of VMH SF-1 neurons ameliorated anxiety-like behavior and suppressed SNS activity. **(A)** Schematic of VMH injection of NpHR AAV viral vector for activation of SF-1 neurons *in vivo*. **(B)** Whole-cell recordings of NpHR-expressing neurons and hyperpolarization (Yellow light, consecutive). **(C)** Representative traces of heart rate change per min evoked by yellow light illumination of VMH SF-1 neurons, and statistical analysis of changes in heart rate (*n* = 5 mice each group, *p* = 0.0038; unpaired *t-*test). **(D)** Representative traces of respiratory rate evoked by yellow light illumination of VMH SF-1 neurons, and statistical analysis of changes in respiratory rate (per min) induced by VMH SF-1 neuron silencing (*n* = 5 mice each group; *p* = 0.3338; unpaired *t*-test). **(E)** Statistical analysis of changes in anxiety-like behavior in OFT induced by 10-min yellow light illumination of VMH SF-1 neurons (*n* = 5 mice each group, Resident time: mCherry, *p* = 0.1687, NPHR, *p* < 0.001; Entries: mCherry, *p* = 0.5161, NPHR, *p* = 0.1402; multiple paired *t*-test). **(F)** Statistical analysis of changes in anxiety-like behavior in EPM induced by optogenetic inhibition of VMH SF-1 neurons (Resident time: mCherry, *p* = 0.9386, NPHR, *p* = 0.0011; Entries: mCherry, *p* = 0.7256, NPHR, *p* = 0.0690; multiple paired *t*-test). Data are means ± SEM, n.s: *P* > 0.05, **P < 0.01, and ***P < 0.001.

We also applied optogenetic inhibition of VMH SF-1 neurons in stressed mice while monitoring their heart rates. By expressing NpHR in the VMH of stressed SF-1 Cre mice, we successfully achieved *in vivo* optogenetic inhibition of VMH SF-1 neurons and simultaneously recorded heart rates (Figures 3A, B). Results showed that 3 min of inhibition of SF-1 neurons acutely decreased the heart rate but not the respiratory rate (Figures 3C, D). To assess the impact of optogenetic suppression of VMH SF-1 neurons on anxiety-like behavior, we conducted open field and EPM tests, which indicated that NpHR-expressing mice were less anxious after yellow light illumination comparing with mCherry-expressing group (Figures 3E, F). These findings suggest that VMH SF-1 neurons can bidirectionally regulate anxiety and sympathetic activation, emphasizing their critical role in linking anxiety states and sympathetic overactivation after chronic stress.

### Excitatory synaptic inputs into the VMH increase after chronic stress

**FIGURE 4:**
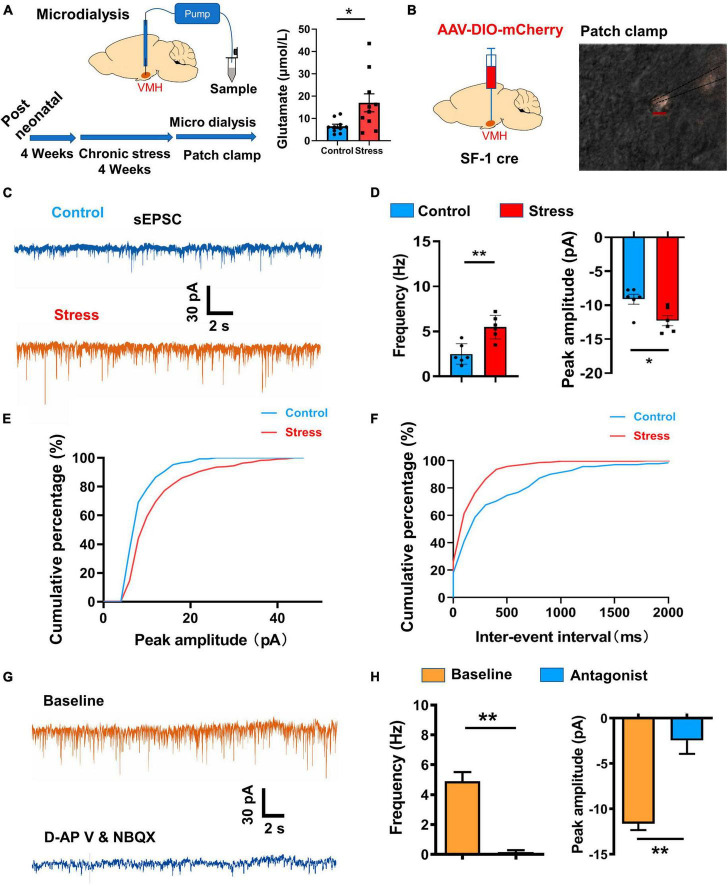
Excitatory synaptic input changes induced by chronic stress in VMH neurons. **(A)** Schematic of chronic stress and microdialysis sampling of VMH neurons *in vivo*, with statistical analysis indicating an obvious increase in glutamate in the VMH (*n* = 8 samples for four control mice and *n* = 10 samples for five stressed mice; *p* = 0.0232; Whitey–Mann U test). **(B)** Schematic of viral injection and patch-clamp recordings of VMH SF-1 neurons *in vitro.* **(C)** Representative traces of sEPSCs (threshold: amplitude > 5 pA) in VMH SF-1 neurons in control and stressed mice. **(D)** Statistical analysis of changes in amplitude and frequency of sEPSC recordings from VMH SF-1 neurons in control and stressed mice (*n* = 6 cells for control and stressed groups, respectively. Amplitude: *p* = 0.0018; Frequency: *p* = 0.0122; unpaired *t*-test). **(E)** Cumulative percentage of sEPSC firing amplitude (pA) curve of VMH neurons from control and stressed mice. **(F)** Cumulative percentage of sEPSC inter-spike interval (ms) curve of VMH neurons from control and stressed mice. **(G)** Effects of glutamate receptor antagonists (d-APV (30 μM) for NMDA receptor and NBQX (20 μM) for AMPA receptor) on sEPSCs of VMH neurons in stressed mice. **(H)** Quantification analysis of changes in sEPSCs (*n* = 5 cells, Amplitude: *p* = 0.0010; Frequency: *p* = 0.0051; paired *t*-test) after applying glutamate receptor antagonists to VMH neurons. Data are means ± SEM, *P < 0.05, and **P < 0.01.

The VMH serves as a critical hub for transmitting and processing stress-related information inputs, thereby playing a regulatory role in the homeostasis of peripheral tissues, such as bone (Yang et al., 2020) and adipose (Viskaitis et al., 2017; Shao et al., 2022). However, the synaptic mechanism responsible for VMH activation following chronic stress remains unclear. To address this issue, we applied microdialysis to measure the concentration of glutamate, an excitatory transmitter, in the VMH. Results revealed a significant increase in glutamate levels in the VMH of the stressed group compared to the control group (Figure 4A).

To further elucidate the underlying electrophysiological mechanism, we conducted whole-cell patch-clamp recordings in the VMH of both stressed and wild-type groups. Specifically, we analyzed spontaneous and miniature excitatory postsynaptic currents (sEPSCs and mEPSCs, holding at – 70 mV) in SF-1 neurons (Figures 4B–D; Supplementary Figure 2), representing the strengthen of excitatory inputs originating from upstream sources. Results demonstrated an increase in both the amplitude and firing frequency of sEPSCs and mEPSCs in the stressed group compared to the control group, suggesting enhancement of excitatory transmission pre- and post-synaptically (Figures 4C–F; Supplementary Figure 2). Subsequently, we sought to identify the receptors responsible for transmitting stress-related information and mediating this enhancement of excitatory inputs. mEPSCs recorded in the existence of TTX (membrane potential holding at - 70 mV) is mainly contributed by AMPA receptor activation (Espinosa and Kavalali, 2009), thus, increase in the amplitude of mEPSCs (Supplementary Figures 2B, C) suggested chronic stress significantly enhanced AMPA receptor-mediated currents in VMH neurons. During sEPSC recordings of stressed group, we applied d-APV and NBQX, highly selective antagonists of glutamate receptors, and observed an obvious suppression of sEPSCs in terms of both firing frequency and amplitude (Figures 4G, H). Together, these findings strongly suggest that glutamate receptors predominantly mediate the enhancement of excitatory inputs in VMH neurons after chronic stress.

### PVN excitatory projections activate the VMH after chronic stress

**FIGURE 5:**
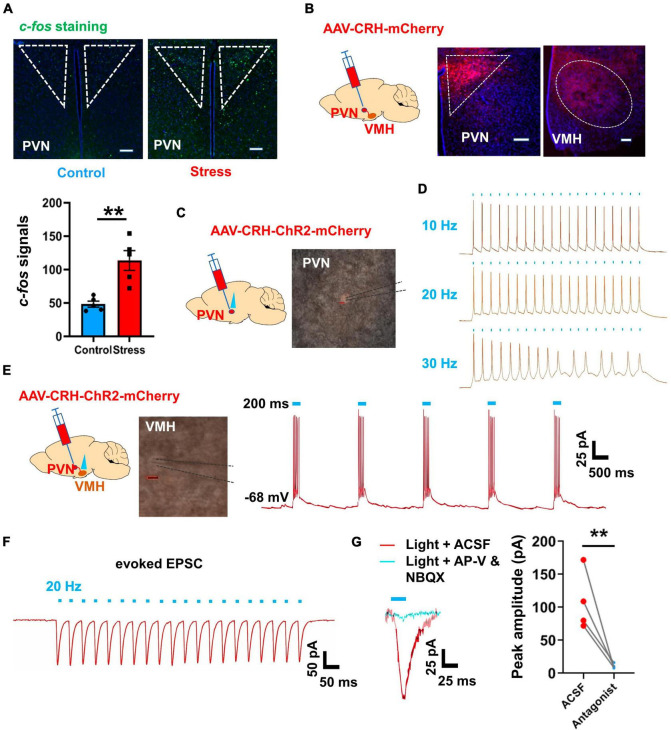
PVN-VMH projections were activated after chronic stress and facilitated VMH neuronal activation. **(A)** *c-fos* staining of PVN in control and stressed groups (scale bar, 100 μm) and quantification of *c-fos*-positive cells in control and stressed groups; (*n* = 5 mice per group, average amount of *c-fos*-positive cells per slice; *p* = 0.0028; unpaired *t*-test). **(B)** Schematic of AAV viral Cre and DIO-mCherry vector injection into PVN to label PVN neurons and their terminals in VMH. **(C)** Schematic of Cre-based and DIO-ChR2-mCherry viral injection into PVN to enable specific ChR2 expression in PVN neurons. **(D)** Whole-cell patch-clamp recordings verified the fidelity of optogenetic manipulation of PVN neurons (10, 20, and 30 Hz, respectively). **(E)** Schematic of DIO-ChR2-mCherry viral injection into PVN to enable specific manipulation of PVN neuronal projections to VMH, and representative traces of action potential firing in VMH neurons elicited by activation of PVN terminals in VMH. **(F)** Evoked EPSCs in VMH neurons induced by optogenetic stimulation of projections from PVN to VMH. **(G)** Evoked EPSCs in VMH neurons were abolished by application of glutamate receptor antagonists (d-APV and NBQX via ACSF prefusion, *n* = 4 cells, paired *t*-test, *p* = 0.0052). Data are means ± SEM, **P < 0.01.

Previous studies have highlighted the critical role of the VMH as a nucleus involved in stress-coping mechanisms (Silva et al., 2016; Shao et al., 2022). In this study, our results indicated that VMH excitatory inputs increased after chronic stress, although the main excitatory upstream source was unclear. Our earlier findings based on retrograde tracing experiments indicated that the BNST, PVN, anterior hypothalamic area, and lateral hypothalamic area are the primary upstream regions projecting into VMH (Yang et al., 2020). Among these nuclei, the PVN is an important acute stress-coping center, especially for the corticotropin-releasing hormone (CRH) expressing subpopulation, which is closely linked to the release of stress-related hormones via the HPA axis (Shenton and Pyner, 2016; Yuan et al., 2019). Our *c-fos* staining results also revealed a greater number of PVN neurons were activated in the chronic stress group comparing with control group (Figure 5A). To further investigate the connectivity between the PVN and VMH, we employed an anterograde tracing approach by injecting an adeno-associated virus (AAV) specifically expressed in CRH-positive neurons. After 4 weeks of expression, we observed terminal signals in the VMH (Figure 5B), consistent with previous study (Yang et al., 2020). Subsequently, we injected AAV-CRH–ChR2-mCherry into the PVN and performed optogenetic manipulation combined with patch-clamp recordings after ChR2 expression *in vitro*. Results showed that optogenetic activation of PVN terminals with blue light evoked action potential firing (Figures 5C–E) and induced strong EPSCs in VMH neurons (Figure 5F). Furthermore, application of glutamate receptor antagonists (d-APV and NBQX) diminished the evoked EPSCs following PVN terminal activation (Figure 5G). Combining these findings with the *c-fos* staining signals (Figure 5A; Supplementary Figure 3), we concluded that the PVN is an important excitatory upstream region that transmits information inputs after chronic stress, thus contributing to the anxiety-like state and associated sympathetic overexcitation.

## Discussion

The complex interconnection between the emotional state in the CNS and maintenance of physiological homeostasis in peripheral tissues is underpinned by the critical mediatory roles of the endocrine system and peripheral SNS (Dallman, 1993; Asakura et al., 2000; Waxenbaum et al., 2023). A growing body of research has emphasized the significance of the VMH as a central hub responsible for the integration of stressful stimuli and control of SNS tone (Kennedy et al., 2020; Zhou et al., 2021; Shao et al., 2022; Liu et al., 2023). At present, however, the electrophysiological foundations and circuitry mechanisms contributing to SNS hyperactivity are yet to be fully elucidated. In the current study, we detected the amplification of sEPSCs and mEPSCs in VMH neurons and identified the PVN as a major source of upstream excitatory inputs to the VMH. Our findings provide strong evidence supporting the role of the VMH as an important hub linking the anxiety state within the CNS and activation of the peripheral SNS. Specifically, chronic stress appears to promote an increase in sympathetic tone outflow by enhancing excitatory input, particularly from the PVN, into the VMH. This dysregulation of the SNS has the potential to disrupt metabolic and cardiovascular homeostasis, thereby contributing to the development of related diseases (Supplementary Figure 4).

### Relation between anxiety and activity of sympathetic nerves

Anxiety is closely associated with peripheral physiological changes, such as increased heart rate and elevated blood pressure, which are critical stress responses essential for the “fight or flight” reaction (Peters et al., 2022). While anxiety in the CNS has evolved to enhance the ability to cope with threats or stressors more efficiently (Abend et al., 2020), there exists a functional correlation between CNS anxiety and stress-related responses in peripheral tissues (Casey et al., 2015; McKim et al., 2016). The SNS mediates outflow from the CNS to innervate peripheral tissues and organs. Various studies have indicated that chronic stress can induce SNS overexcitation, characterized by enhanced electrophysiological activity and increased release of NE from sympathetic nerve endings (Goddard et al., 2010; Fonkoue et al., 2018; Holwerda et al., 2018). Research has also established the existence of SNS nerves in several peripheral tissues and organs, such as liver, adipose, bone, and kidney (Amann and Constantinescu, 1990; Nguyen et al., 2014; Yang et al., 2020; Torres et al., 2021). These nerves and innervated organs can be affected by chronic stress and anxiety, leading to disruptions in physiological homeostasis. For instance, individuals with panic disorder may experience an anxiety-induced increase in relative burst amplitude of sympathetic nerve activity in their muscles (Holwerda et al., 2018). Clinical evidence also strongly suggests that SNS overexcitation can directly and indirectly contribute to elevated blood pressure, often through RAAS or SNS nerve stimulation within the kidney (Bangsumruaj et al., 2022), and to a heightened risk of type 2 diabetes (Sharma and Singh, 2020) and obesity (Tamashiro et al., 2011). However, the precise mechanism by which critical hubs within the CNS regulate chronic stress-induced anxiety and modulate SNS activity toward a hyperexcited state still require further exploration.

### VMH is critical in both chronic stress-induced anxiety and peripheral SNS innervation

The transmission and processing of negative stress information within the brain involve complex neural circuits, ultimately leading to the induction of negative emotional states such as like anxiety. Several key nodes within stress-related neural circuits have been identified, including the amygdala, BNST, and lateral hypothalamus (Choi et al., 2007; Anthony et al., 2014). Recent research has highlighted the essential role of the VMH in mediating chronic stress-induced anxiety-like behaviors and encoding stress-related emotional states (Silva et al., 2017; Shao et al., 2022). In addition to its involvement in anxiety regulation, the VMH is also considered a critical modulator of energy homeostasis in the peripheral system (Dhillon et al., 2006; Sohn et al., 2016). In our previous research, we found that stress-induced changes in VMH neural activity can impact the peripheral SNS and regulate bone metabolism (Yang et al., 2020). In the current study, we found that long-term repeated stress influenced excitatory neurotransmission (including increased glutamate release from presynaptic terminals and enhanced AMPA receptor-mediated postsynaptic currents) into the VMH from upstream regions, thereby affecting neuronal excitability and action potential firing. These electrophysiological changes induced anxiety-like behavior and changes in SNS tone, consequently regulating several peripheral physiological processes. Many research indicated rostral raphe pallidus (RPa), a downstream of VMH projection, is involved in regulating SNS activity and heart rate (Cao and Morrison, 2003; Wang et al., 2018). Thus, activation of VMH caused by chronic stress could enhance the excitability of RPa neurons and increase heart rate, while inhibiting VMH neuronal activity under chronic stress may attenuate this innervation and decrease hear rate. Furthermore, our previous study revealed enhanced inhibitory postsynaptic currents in VMH neurons after chronic stress, which triggered anxiety-like behavior and bone loss via the SNS (Yang et al., 2020). Subsequent investigations also indicated that the inhibitory inputs may induce “rebound” burst firing of VMH neurons, contributing to the regulation of anxiety and related energy expenditure imbalance (Yang et al., 2020; Shao et al., 2022). Collectively, these findings imply that the VMH receives and processes chronic stress information from upstream brain regions, such as the PVN, while also regulating SNS outflow. Thus, the VMH serves as a critical link that establishes a significant relationship between chronic stress-induced anxiety and SNS overexcitation, ultimately promoting stress-related physiological imbalance in peripheral tissues. However, further exploration is required to understand how chronic stress strengthens the synaptic connections between the PVN and VMH and sustains this increase. Moreover, further studies are necessary to clarify the contributions of projections from other regions, such as the anterior hypothalamic area and lateral hypothalamic area, to the observed enhancement of sEPSCs. In the current study, we primarily focused on elucidating the crucial role of the VMH in regulating anxiety and associated sympathetic activity in male mice under chronic stress. Thus, further research is required to determine whether these findings are sex dependent.

## Conclusion

In this study, we demonstrated the pivotal role of the VMH in mediating chronic stress-induced anxiety and SNS activity. Importantly, our findings indicated that optogenetic stimulation of VMH SF-1 neurons resulted in anxiety-like behavior and peripheral sympathetic arousal, while suppressing these neurons yielded the opposite effects. We also showed that chronic stress amplified the excitatory inputs received by VMH neurons, thereby escalating anxiety and elevating sympathetic tone, which significantly contributed to the induction of metabolic imbalance in peripheral tissues. Furthermore, we identified the PVN as a primary node receiving a wide range of external stress signals and internal physiological cues to effectively regulate physiological stress responses. The PVN, populated with a variety of stress-responsive neuronal types, prominently featured densely distributed CRH-expressing neurons, which played crucial roles in the stress response. Our tracing data revealed that CRH-expressing neurons in the PVN projected to the VMH and sufficiently activated VMH neurons under conditions of chronic stress. These findings broaden our understanding of the relationship between chronic stress-induced anxiety and SNS hyperexcitation, offering new potential targets for interventions aimed at treating chronic stress-induced anxiety and associated physiological imbalances in peripheral tissues.

## Data availability statement

The original contributions presented in this study are included in this article/Supplementary material, further inquiries can be directed to the corresponding authors.

## Ethics statement

The animal study was approved by the Ethics Committee of the Animal Care and Use Committee of Shenzhen People’s Hospital and Shenzhen Institutes of Advanced Technology, Chinese Academy of Sciences. The study was conducted in accordance with the local legislation and institutional requirements.

## Author contributions

JS: Data curation, Formal analysis, Funding acquisition, Writing—original draft, Writing—review and editing. YC: Investigation, Methodology, Writing—original draft. DG: Investigation, Methodology, Writing—original draft. YL: Funding acquisition, Methodology, Writing—original draft. NH: Funding acquisition, Methodology, Writing—original draft. LY: Writing—review and editing. XZ: Funding acquisition, Supervision, Writing—original draft, Writing—review and editing. FY: Funding acquisition, Supervision, Writing—original draft, Writing—review and editing.

## Supplementary material

Supplementary Figure 1 Pearson correlation analysis between anxiety-like behavior and concentrations of stress-related hormones. **(A,B)** Pearson correlation analysis between anxiety-like behavior and concentration of cortisol: Left, correlation between resident time in central area of open field and cortisol concentration, *R* = −0.841, *p* < 0.01; Right, correlation between resident time in open arms of EPM and cortisol concentration, *R* = −0.608, *p* = 0.021. **(C,D)** Pearson correlation analysis between anxiety-like behavior and concentration of renin: Left, resident time in central area of open field and renin concentration, *R* = −0.657, *p* = 0.011; Right, resident time in open arms of EPM and renin concentration, *R* = −0.416, *p* = 0.139 (*n* = 7 for each group).

Supplementary Figure 2 Enhanced mEPSCs in VMH after chronic stress. **(A)** Representative traces of mEPSCs (threshold: amplitude > 5 pA, holding at - 70 mV with 1 μM TTX) in VMH SF-1 neurons in control and stressed mice. **(B)** Statistical analysis of changes in amplitude and frequency of mEPSC recordings from VMH SF-1 neurons in control and stressed mice (*n* = 6 cells for control and stressed groups, respectively. Amplitude: *p* = 0.018; Frequency: *p* = 0.0192; unpaired *t*-test). **(C)** Cumulative percentage of mEPSC firing amplitude (pA) curve of VMH neurons in control and stressed mice. **(D)** Cumulative percentage of mEPSC inter-spike interval (ms) curve of VMH neurons in control and stressed mice.

Supplementary Figure 3 *c-fos* immunostaining of BNST, anterior and lateral hypothalamic regions. **(A)** Representative images of *c-fos* staining of BNST in control and stressed groups (scale bar, 100 μm) and quantification of *c-fos*-positive cells in control and stressed groups; (*n* = 5 mice per group, average amount of *c-fos*-positive cells per slice; *p* < 0.001; unpaired *t*-test). **(B)** Quantification of *c-fos* signals of lateral hypothalamus (LH) and anterior hypothalamus (AHC) **(C)** in control and stressed groups (*n* = 5 mice per group, average amount of *c-fos*-positive cells per slice; LH, *p* = 0.0340; AHC, *p* = 0.7934, unpaired *t*-test; scale bar: 100 μm).

Supplementary Figure 4 VMH SF-1 neurons transmitted chronic stress inputs and exerted bidirectional regulation on anxiety state and sympathetic outflow. PVN, paraventricular hypothalamus; VMH, ventromedial hypothalamus; EPSC, excitatory postsynaptic current; SNS, sympathetic nervous system; CRH, corticotropin-releasing hormone; SF-1, steroidogenic factor-1.
