# Supplementary material for: Ventromedial hypothalamus relays chronic stress inputs and exerts bidirectional regulation on anxiety state and related sympathetic activity
Source: Front Cell Neurosci. 2023 Dec 14;17:1281919. doi: 10.3389/fncel.2023.1281919 (PMC10755867; doi:10.3389/fncel.2023.1281919)
Supplement: Supplementary Figure 1 — Pearson correlation analysis between anxiety-like behavior and concentrations of stress-related hormones. (A,B) Pearson correlation analysis between anxiety-like behavior and concentration of cortisol: Left, correlation between resident time in central area of open field and cortisol concentration, R = −0.841, p < 0.01; Right, correlation between resident time in open arms of EPM and cortisol concentration, R = −0.608, p = 0.021. (C,D) Pearson correlation analysis between anxiety-like behavior and concentration of renin: Left, resident time in central area of open field and renin concentration, R = −0.657, p = 0.011; Right, resident time in open arms of EPM and renin concentration, R = −0.416, p = 0.139 (n = 7 for each group). [file Image_1.pdf]

Supplementary Material

Ventromedial hypothalamus relays chronic stress inputs and exerts bidirectional regulation on anxiety state and related sympathetic activity

Jie Shao [1,2], Yan Chen [3,4], Dashuang Gao [3], Yunhui Liu [3], Nan Hu [5], Lianghong Yin [2], Xinzhou Zhang [5*], Fan Yang [3*].

Supplementary Figures

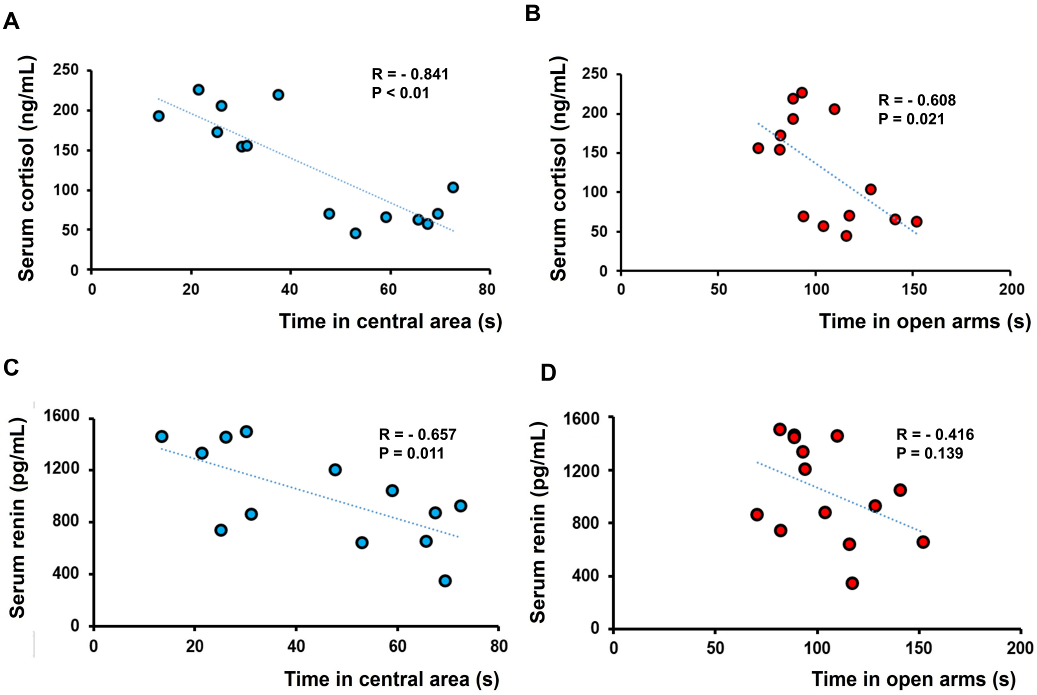

Supplementary Figure 1. Pearson correlation analysis between anxiety-like behavior and concentrations of stress-related hormones.

(**A** and **B**) Pearson correlation analysis between anxiety-like behavior and

concentration of cortisol: Left, correlation between resident time in central area of open field and cortisol concentration, R = - 0.841, $p < 0.01$; Right, correlation between resident time in open arms of EPM and cortisol concentration, R = - 0.608, $p = 0.021$. (**C** and **D**) Pearson correlation analysis between anxiety-like behavior and concentration of renin: Left, resident time in central area of open field and renin concentration, R = - 0.657, $p = 0.011$; Right, resident time in open arms of EPM and renin concentration, R = -0.416, $p = 0.139$. (n = 7 for each group)

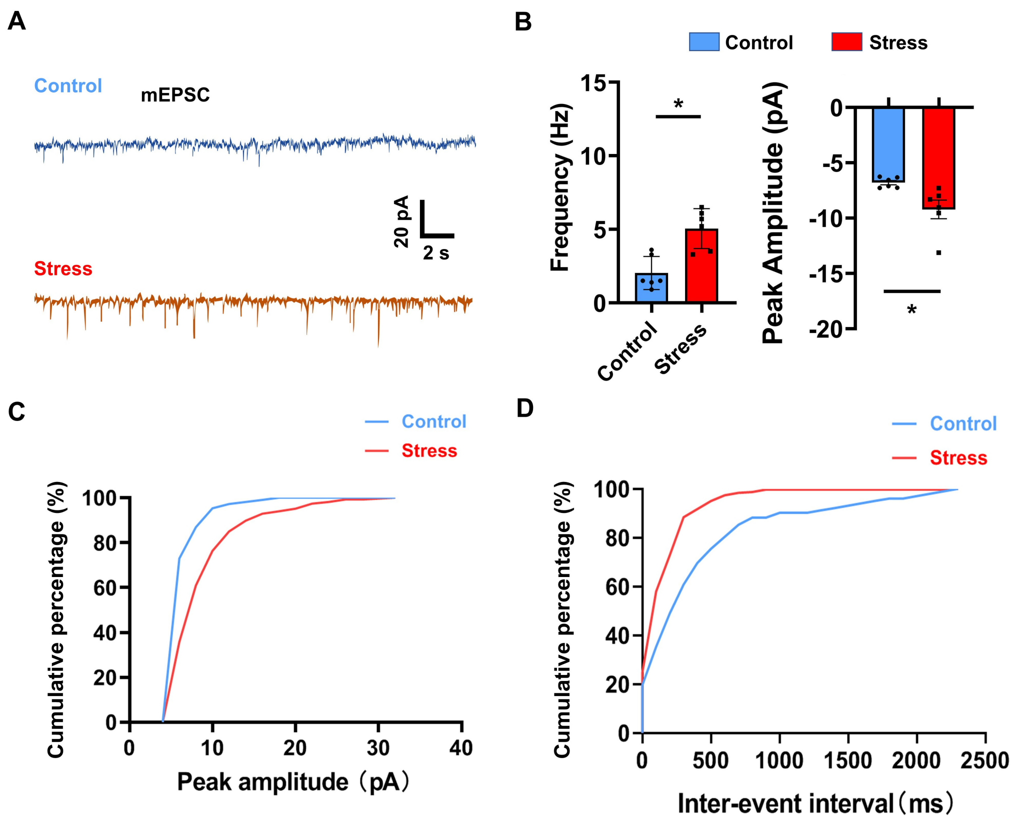

Supplementary Figure 2. Enhanced mEPSCs in VMH after chronic stress.

(A) Representative traces of mEPSCs (threshold: amplitude > 5 pA) in VMH SF-1 neurons in control and stressed mice. **(B)** Statistical analysis of changes in amplitude and frequency of mEPSC recordings from VMH SF-1 neurons in control and stressed mice (n = 6 cells for control and stressed groups, respectively. Amplitude: p = 0.018; Frequency: p = 0.0192; unpaired t-test). **(C)** Cumulative percentage of mEPSC firing amplitude (pA) curve of VMH neurons in control and stressed mice. **(D)** Cumulative percentage of mEPSC inter-spike interval (ms) curve of VMH neurons in control and stressed mice. *p < 0.05, **p < 0.01.

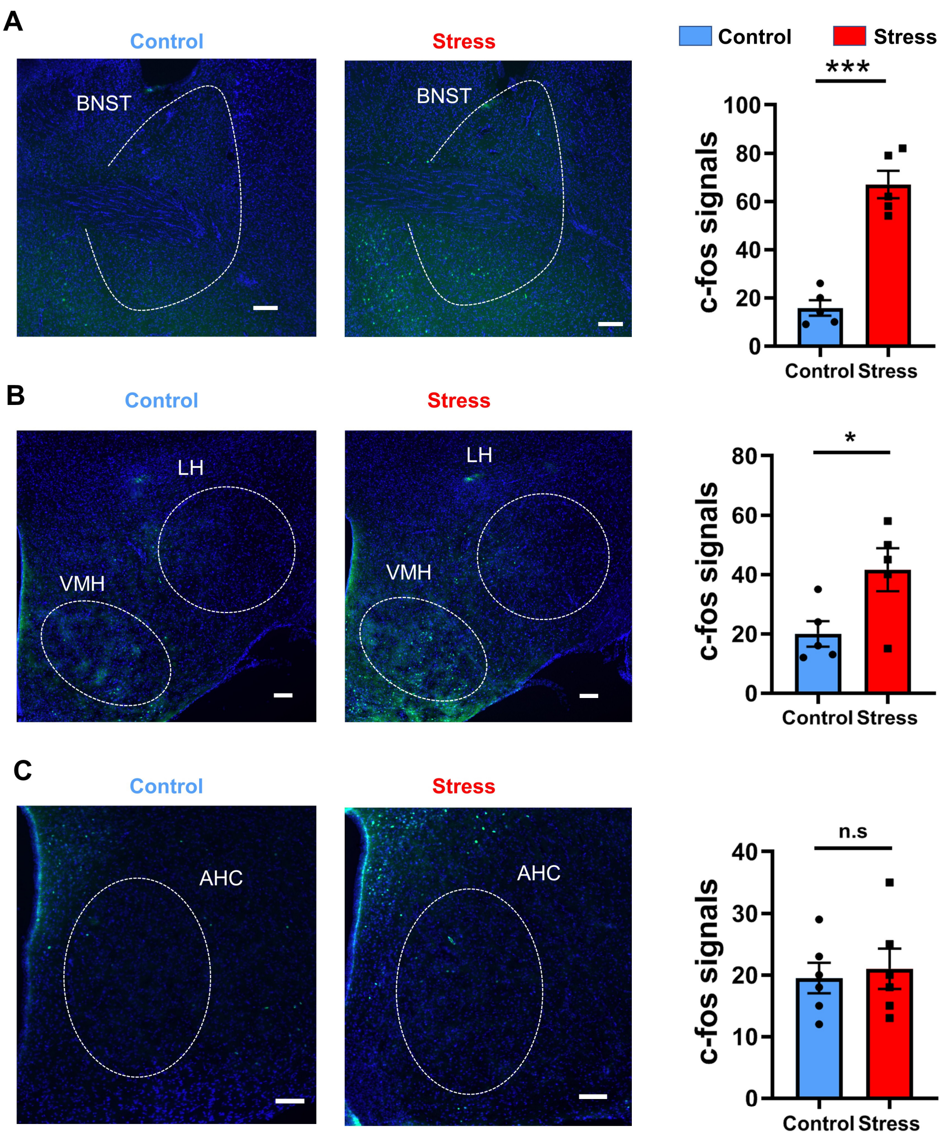

Supplementary Figure 3. ***c-fos*** **immunostaining of BNST, anterior and lateral hypothalamic regions.**

(A) Representative images of *c-fos* staining of BNST in control and stressed groups (scale bar, 100 μm) and quantification of *c-fos*-positive cells in control and stressed groups; (n = 5 mice per group, average amount of *c-fos*-positive cells per slice; $p < 0.001$; unpaired *t*-test). **(B)** Quantification of *c-fos* signals of lateral hypothalamus (LH) and anterior

hypothalamus (AHC) **(C)** in control and stressed groups (n = 5 mice per group, average amount of *c-fos*-positive cells per slice; LH, $p = 0.0340$; AHC, $p = 0.7934$, unpaired *t*-test; scale bar: 100 μm). n.s: $p > 0.05$, *$p < 0.05$, **$p < 0.01$, ***$p < 0.001$.

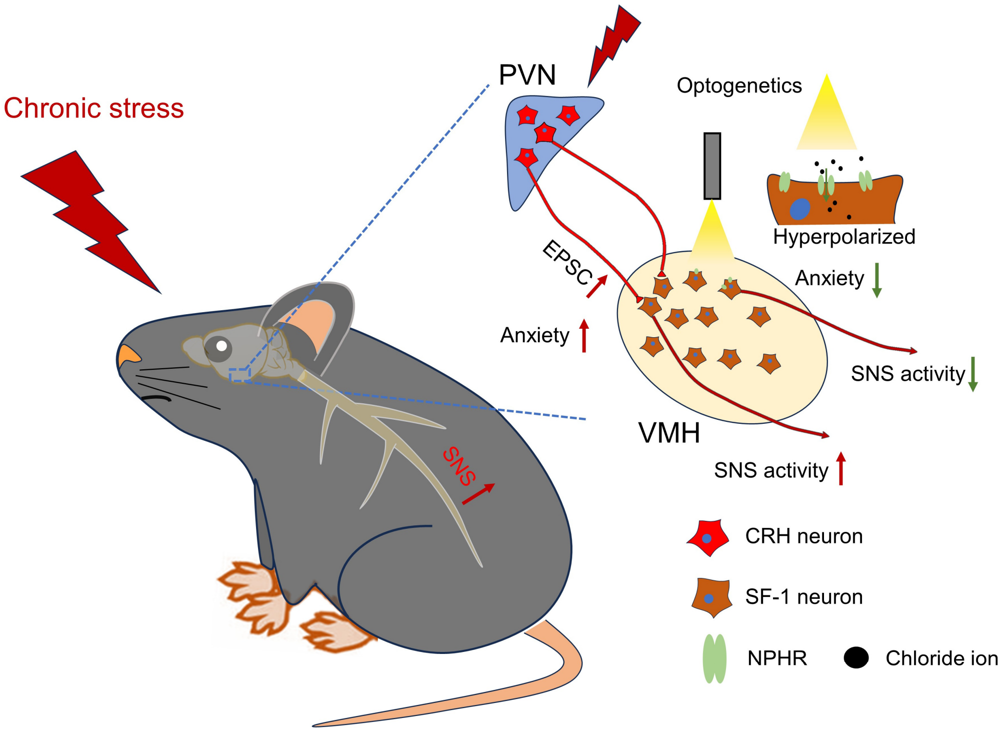

Supplementary Figure 4. VMH SF-1 neurons transmitted chronic stress inputs and exerted bidirectional regulation on anxiety state and sympathetic outflow. PVN: paraventricular hypothalamus; VMH: ventromedial hypothalamus; EPSC: excitatory postsynaptic current; SNS: sympathetic nervous system; CRH: corticotropin-releasing hormone; SF-1: steroidogenic factor-1.
